# A Systematic Review of Suggested Molecular Strata, Biomarkers and Their Tissue Sources in ALS

**DOI:** 10.3389/fneur.2019.00400

**Published:** 2019-05-14

**Authors:** Udaya Geetha Vijayakumar, Vanessa Milla, Mei Yu Cynthia Stafford, Anthony J. Bjourson, William Duddy, Stephanie Marie-Rose Duguez

**Affiliations:** Northern Ireland Center for Stratified Medicine, Biomedical Sciences Research Institute, Londonderry, United Kingdom

**Keywords:** circulating biomarkers, ALS, patients stratification, multi-system biomarkers, motor neuron disease

## Abstract

Amyotrophic lateral sclerosis (ALS), also known as motor neuron disease, is an incurable neurodegenerative condition, characterized by the loss of upper and lower motor neurons. It affects 1–1.8/100,000 individuals worldwide, and the number of cases is projected to increase as the population ages. Thus, there is an urgent need to identify both therapeutic targets and disease-specific biomarkers–biomarkers that would be useful to diagnose and stratify patients into different sub-groups for therapeutic strategies, as well as biomarkers to follow the efficacy of any treatment tested during clinical trials. There is a lack of knowledge about pathogenesis and many hypotheses. Numerous “omics” studies have been conducted on ALS in the past decade to identify a disease-signature in tissues and circulating biomarkers. The first goal of the present review was to group the molecular pathways that have been implicated in monogenic forms of ALS, to enable the description of patient strata corresponding to each pathway grouping. This strategy allowed us to suggest 14 strata, each potentially targetable by different pharmacological strategies. The second goal of this review was to identify diagnostic/prognostic biomarker candidates consistently observed across the literature. For this purpose, we explore previous biomarker-relevant “omics” studies of ALS and summarize their findings, focusing on potential circulating biomarker candidates. We systematically review 118 papers on biomarkers published during the last decade. Several candidate markers were consistently shared across the results of different studies in either cerebrospinal fluid (CSF) or blood (leukocyte or serum/plasma). Although these candidates still need to be validated in a systematic manner, we suggest the use of combinations of biomarkers that would likely reflect the “health status” of different tissues, including motor neuron health (e.g., pNFH and NF-L, cystatin C, Transthyretin), inflammation status (e.g., MCP-1, miR451), muscle health (miR-338-3p, miR-206) and metabolism (homocysteine, glutamate, cholesterol). In light of these studies and because ALS is increasingly perceived as a multi-system disease, the identification of a panel of biomarkers that accurately reflect features of pathology is a priority, not only for diagnostic purposes but also for prognostic or predictive applications.

## Introduction

Amyotrophic lateral sclerosis (ALS) is a fatal neurological disorder with an adult onset around 54–67 years old (1). Its clinical hallmark is the degeneration of both upper and lower motor neurons (2, 3), leading to progressive muscle atrophy and weakness, and ultimately to paralysis. Death, often resulting from swallowing problems and respiratory failure (4, 5), generally occurs within 2–4 years from disease onset (6–8), although 5–10% of ALS patients survive over 10 years (7). ALS has a median incidence of about 2.8 cases per 100,000 persons per year and a median prevalence about 5.4 cases per 100,000 persons for a median age at 61.8 ± 3.8 years (1). The incidence and prevalence thus increases with age and reaches a cumulative lifetime risk of 1 in 400 after 80 years old (9, 10). Due to the projected aging of the global population, ALS cases are expected to increase by 69% in the next 25 years (11), underlining the urgent need to identify causes, biomarkers and therapeutic targets for ALS.

**Figure 1:**
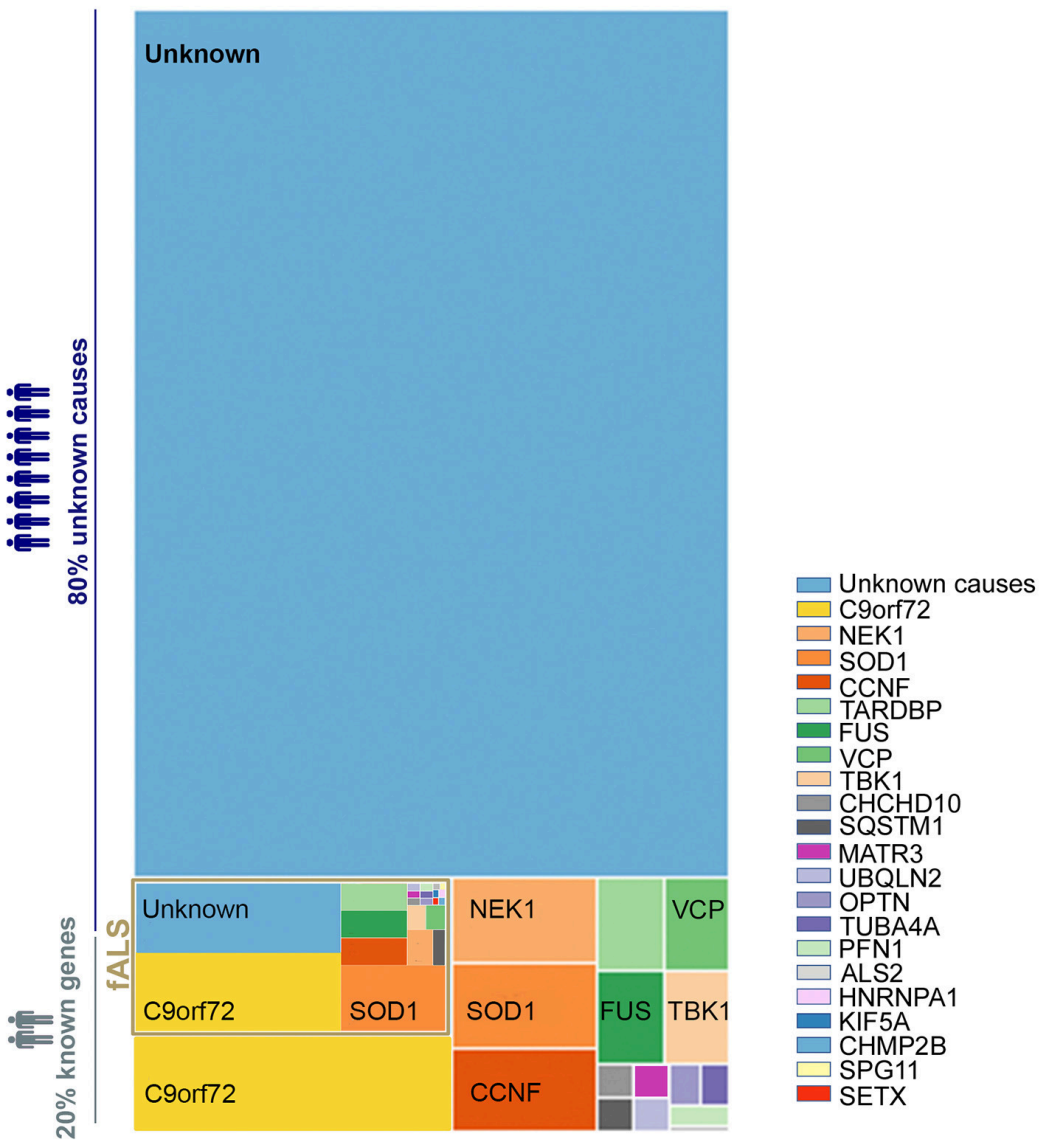
Distribution of genetic basis among the ALS population. A treemap representation of the proportion of ALS patients carrying known causative mutation. The full rectangle represents 100% of all ALS cases. The fALS are highlighted in gold with a frequency adjusted to represent 7.5% of the total (as fALS is estimated at 5–10% of all ALS cases). The two light blue blocks represent those with no known ALS-associated gene mutation among sporadic and familial cases. Cases with known mutations are represented in the other blocks, broken down by affected gene. The color code for each gene is preserved between familial and sporadic cases. The size of each block is proportional to the percentage of ALS associated to the considered genes–proportions given in Volk et al. (13). Overall, some 80% of ALS cases (sALS and fALS combined) are not explained by a known mutation.

**Figure 2:**
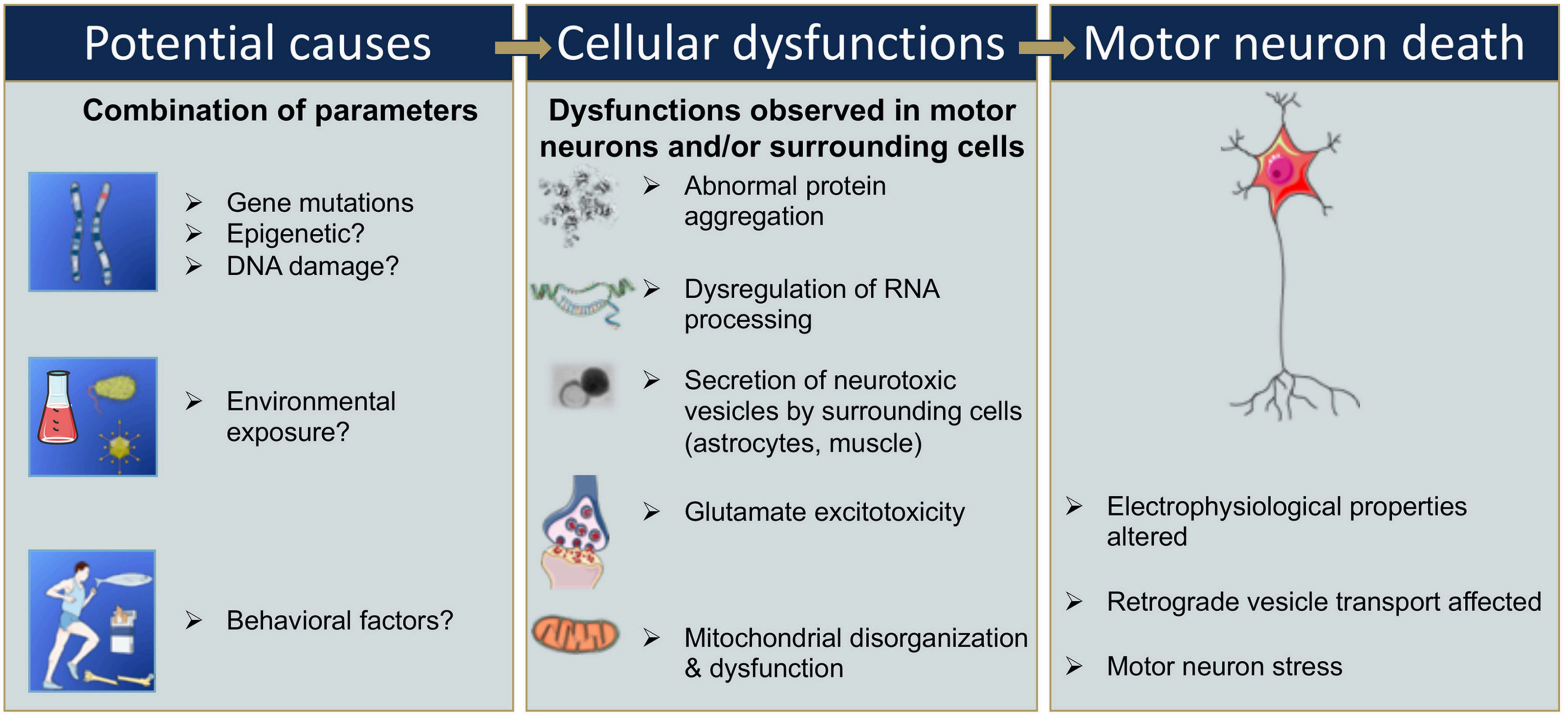
Sequential events that could be involved in motor neuron death in ALS. Gene mutations, epigenetic changes, or DNA damage that occur either spontaneously or due to environmental risk factors such as exposure to toxins or infectious agents, or behavioral factors, have all been proposed as potentially leading to cellular dysfunction (9, 13, 14, 20–23). Cellular dysfunction could include abnormal protein aggregations, alteration of RNA processing, secretion of neurotoxic vesicles by surrounding cells such as astrocyte, muscle cells, glutamate excitotoxicity, and mitochondrial disorganization and dysfunction leading to oxidative stress (24–30). These cellular dysfunctions may take place in motor neurons and/or surrounding cells and, combined or alone, could lead to an alteration of the electrophysiological properties of the motor neuron, and/or to an induction of secretion of neurotoxic elements by surrounding cells, in either case ultimately leading to motor neuron death (16–18).

The causes of ALS are largely unknown, with ~90% of cases being sporadic (sALS) while only ~10% are familial ALS (fALS) (12). Intensive research since the 1990's has aimed to unravel the mechanisms involved in motor neuron degeneration. These studies suggest that ALS is a complex disease driven by a combination of several systemic parameters. To date, up to 30 genes (Figure 1) are described as monogenic causes of ALS, with the most frequent being C9orf72, SOD1, FUS, and TARDBP/TDP43 (13–15). In motor neurons, these identified mutations are functionally associated with an alteration of electrophysiological properties (16), accumulation of stress marks (17) and sensitivity to stress (18) (Figure 2). However, these monogenic forms explain only 15% of sporadic cases and 66% of familial cases (12) (Figure 1).

Furthermore, the penetrance of these disease-associated mutations is quite variable and can increase with age (12, 19). The variability in penetrance as well as the lack of identification of a single associated gene mutations in 85% of sALS suggests that some ALS cases have a multigenic component, and/or involve epigenetic modification, and/or result from DNA damage, environmental risk factors, or viral infections (9, 14, 20–23) (Figure 2). In these cases, it is likely a combination of these factors that leads to cellular dysfunction such as glutamate-mediated excitotoxicity (24), abnormal protein aggregation (25), mitochondrial disorganization and dysfunction (26, 27) contributing to the oxidative stress (28–30) (Figure 2). Adding to the complexity of ALS, several studies suggest that not only the motor neurons are affected but also the surrounding cells, and that these cells participate in the propagation and burden of the disease. For instance, activated microglia cells release superoxide and nitric oxide metabolites, elements that are toxic to neuronal cells (31). Astrocytes can also participate in the propagation of neurotoxic elements (32, 33) such as SOD1 aggregates (34–36), and a failure of astrocytes to remove extracellular glutamate may mediate excitotoxicity (37–39). Ultimately, the intracellular dysfunction of the motor neuron combined with aberrant secretion of neurotoxic elements of surrounding cells leads to motor neuron stress, aberrant electrophysiological properties, and consequently to motor neuron death (Figure 2).

In the absence of a reliable diagnostic test for ALS, diagnosis is based on clinical and electrophysiological criteria such as evidence for progressive involvement of both upper and lower motor neurons and exclusion of diseases mimicking ALS as set out in the Revised El Escorial Criteria (REEC), Airlie House criteria (AHC) and Awaji criteria (2, 40). The process of diagnosis can be lengthy and there is a typical diagnostic delay of 9-15 months from onset to diagnostic confirmation (41). Considering that the average survival from onset is 2–4 years (6–8) and that efficacy of Riluzole is improved by early treatment (42), there is an urgent need to improve diagnostic speed and accuracy for ALS. One way of achieving this is the identification of biomarkers specific to ALS pathology, to enable the development a reliable fast diagnostic test. As well as diagnostics, it is also important to identify prognostic biomarkers that can be used to monitor the status of the pathology–various candidates may serve both these purposes. The identification of ALS biomarkers will contribute to a better understanding of the disease pathogenesis, and permit targeted drug development and patient stratification for more efficient clinical trials, assuming that different sub-cohorts of ALS patients respond differently to treatments. Biomarker discovery can be achieved by examining the “omics” contents of ALS patient tissues.

The present review has two aims: (1) to identify pathways commonly affected in genetic forms of ALS, and stratify the patients accordingly, and (2) to explore previous genomic, transcriptomic, proteomic, metabolomic and miRNomic studies of ALS published during the last decade, and summarize the findings, highlighting potential biomarker candidates for ALS disease management and treatment.

### Genetic Markers for ALS Patient Stratification

The first gene identified to be associated with ALS was SOD1 in 1993 (43). Since then 29 new genes have been identified (13–15), representing the most frequent genetic mutations included in current diagnostic processes (13, 44) (Figure 1). These 30 genes offer crucial clues in understanding the pathogenesis of ALS—some of the gene products interact with each other (14)—and enable the identification of diverse cellular pathways that are disrupted in ALS patients (Table 1). Even if most ALS cases are sporadic, the pathways disrupted in familial cases may also be affected in sporadic cases, as both sALS and fALS can share common molecular signatures or functional biological effects such as FUS or TDP43 protein aggregations or accumulation of stress granules formation (45), disruption in RNA processing (46), or disruption of autophagy and mitochondrial functions (47). When sorting the genes associated to ALS according to their primary cellular functions, several categories of dominantly affected pathway can be highlighted, such as (1) mitochondrial metabolism and turnover, (2) axonal transport and the cytoskeleton, (3) autophagy and proteostasis, (4) endosomal and vesicular trafficking, (5) DNA repair, and (6) ribostasis/RNA alteration/Nucleocytoplasmic transport—with most of the genes being involved in multiple pathways. It may be possible to group patients into strata depending on which combination of pathways is dysregulated, and to recruit patients accordingly for translational research and clinical trials. We have cautiously assigned each causal gene to one of 14 strata, depending on the profile of its affected pathways (Table 1). These groupings represent our effort to summarize current understanding and are not intended to be definitive—indeed, it will be important to modify and update them on an ongoing basis with improvements in the knowledge of protein function and the impact of mutations. Although these 14 strata are directly applicable to only 20% of total ALS cases (Figure 1), future work may determine whether (and which of) these molecular signatures are implicated in the remaining cases.

**Table 1:**
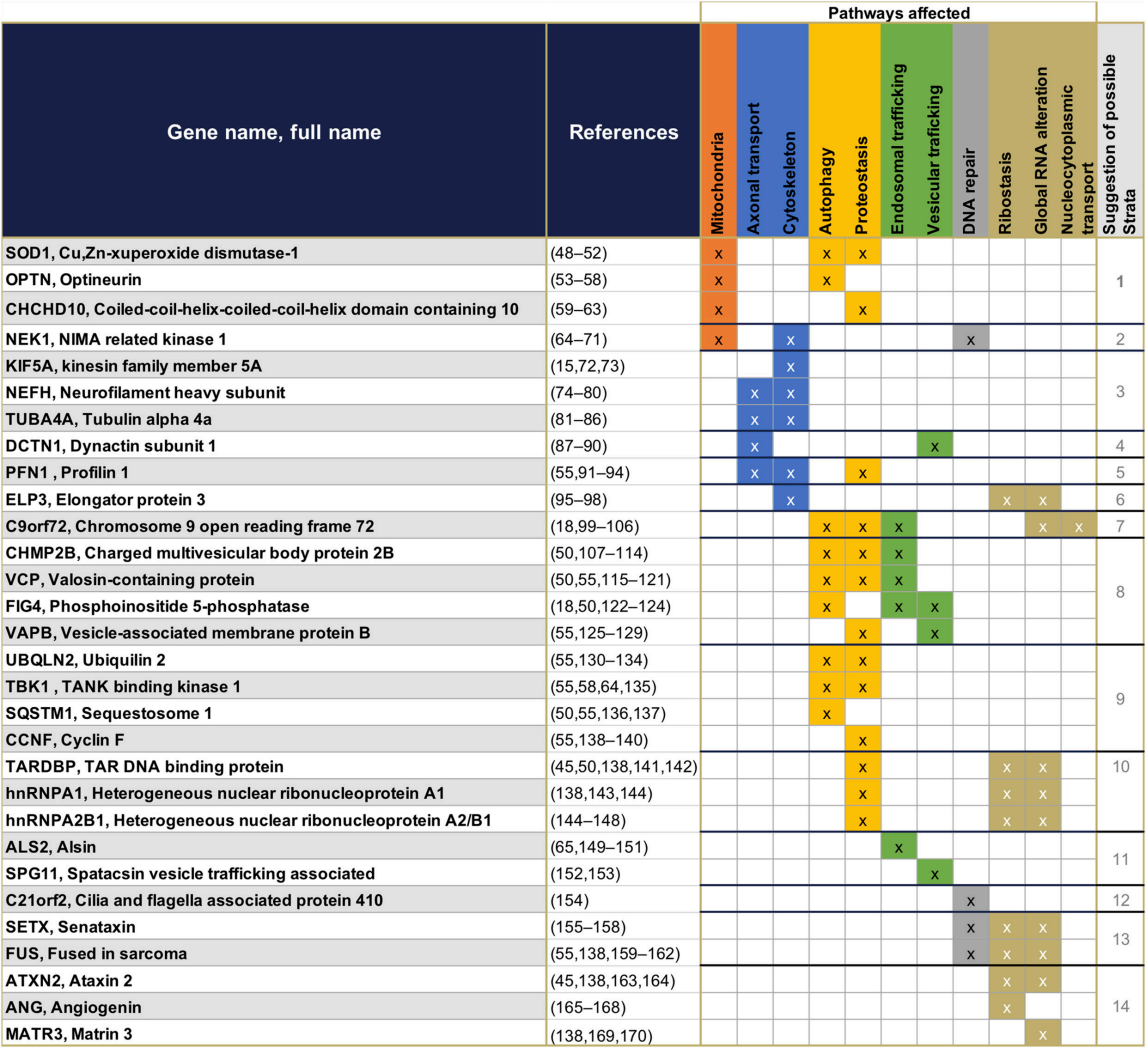
Summary of the 30 genes presently known to have monogenic association with ALS, and their primary functions.

*The list of genes is taken from Volk et al. (13) and Chia et al (14). The references given in the second column indicate papers providing experimental evidence of the primary pathways (or molecular functions) affected in cell and animal models harboring the respective mutation. These pathways are given in columns 3-13–it should be noted that our understanding of the implicated pathways may change in future as more is known regarding the effects of mutations. We grouped together pathways commonly affected across genetic forms of ALS, and we suggest 14 potential strata based on the profiles of affected pathways. These groupings represent our effort to summarize current understanding and are not intended to be definitive–indeed, it will be important to modify and update them on an ongoing basis as the knowledge of protein loss and gain of function improves*.

### The Search for Circulating Biomarkers

The identification of circulating markers associated with ALS pathology would be important tools to provide early disease diagnosis and to track progression or treatment. There has been a concerted focus aimed at identifying such biomarkers in different body fluids over the past 20 years. In Table S1, we summarized 76 studies that investigated proteins, miRs, mRNAs, and metabolites as potential biomarkers in cerebrospinal fluid (CSF) or blood (blood cells, serum or plasma). To date, little has been done investigating urine-based biomarkers, and thus urine biomarker analyses are not reported in the current review. CSF is the most frequently used sample source, and several studies (Table S1) report a consistent decrease in protein levels of transthyretin—involved in neurogenesis, nerve repair and axonal growth (171)—and cystatin c—an endogenous cysteine protease inhibitor that can protect motor neurons against neurotoxicity by stimulating autophagy and inhibition of cathepsin B (172). In addition, CSF cystatin C protein levels positively correlated with the survival of ALS patients and could be thus potentially used as a prognostic biomarker (173). However, both transthyretin and cystatin C decreases are not specific to ALS patients and a similar pattern is observed in other neurodegenerative diseases (173) such as Alzheimer's (171), suggesting that the protein levels of both transthyretin and cystatin C level are a common signature for neuron vulnerabilities and neurodegeneration. The protein levels of neurofilament light chain (NF-L) and the phosphorylated form of neurofilament heavy chain (pNFH) were also consistently found to be increased in the CSF of ALS patients across multiple studies (Table S1), with a high level of either NF-L or pNFH predicting a shorter life expectancy (174–178). NF-L and pNFH are markers for axonal damage (179). In this context, similarly to M-creatine kinase for myofiber fragility in muscular dystrophy (180), NF-L and pNFH thus directly reflect the health of the neurons –the cells specifically impacted by ALS.

Combining NF-L and pNFH with other markers that reflect the “health status” of other tissues such as glial cells, skeletal muscle, or inflammatory response, may represent a useful addition, as ALS is now perceived as a multisystemic disease. Such a multi-marker approach may represent a useful complement to a panel of biomarkers to test the efficacy of drugs in clinical trials. In this respect, miR-451—an inhibitor of microglial cell activation (181)—was consistently decreased in leukocytes of ALS patients (Table S1), while the pro-inflammatory MCP-1, secreted by the glial cells and neurons (182), was found to be increased in both serum and plasma (Table S1). Both miR-451 and MCP-1 could thus potentially inform the status of inflammatory cell recruitment and activation (181, 182). In addition, miR-206, which is essential for skeletal muscle growth and regeneration (183), as well as miR-338-3p, a regulator of neuromuscular junctions (184), are consistently upregulated in leukocytes—with miR-206 also consistently reported to be upregulated in serum and plasma samples across multiple studies (Table S1). In this context, miR-206 and miR-338-3p could be clinically useful candidate biomarkers of the health status of skeletal muscle (185).

Regarding circulating mRNAs, no obvious consistent candidates have been identified yet across previous studies (Table S1). With regard to analyses of circulating metabolite candidates, huge variation is observed between studies, though there was a general tendency for upregulation of specific metabolites in serum and plasma (Table S1), which is consistent with the hypermetabolism observed in some ALS patients (186). For instance, creatine, which is linked to cell energy metabolism, was consistently increased in CSF and plasma across studies (Table S1). Pyruvate and glucose were also found to be increased in CSF and plasma of ALS patients (Table S1), potentially reflecting a dysregulation of glycolytic metabolism as observed in SOD1-G93A motor neurons (187), and in some ALS cases (188, 189). This upregulation of glycolysis correlates with a shorter survival time and thus could be used as a prognostic biomarker (188, 189). Similarly, the upregulation of cholesterol and LDL observed in CSF and plasma across studies (Table S1) could also reflect a global dysregulation of lipid metabolism in ALS patients (190, 191). Other neurotoxic metabolites, such as homocysteine, were consistently increased in all body fluids (Table S1). Altogether, these data suggest a global dysregulation of the energy metabolism in ALS patients.

Other types of molecules could be investigated as biomarkers in ALS, such as long non-coding RNA (lncRNA), which can act in cis to either silence or enhance the expression of proximal genes (192) and which are known to have a key role in normal neuronal development, as well as in development and progression of neurodegenerative diseases [see (193) for review]. The lncRNA have also been detected in body fluids and have been suggested as potential diagnostic and/or prognostic biomarkers in, but not only, lung cancer (194), triple negative breast cancer (195) and cardiovascular diseases (196). In this context, lncRNA could be investigated as new biomarker candidates for neurodegenerative diseases (193), including ALS.

## Exploring potential ALS Signatures in Tissue

Studying changes at the molecular level of specific tissues affected in ALS should improve our understanding of the disease mechanisms and multi-systemic impact.

Postmortem brain or spinal cord have been widely investigated. Accumulation of pNF-H and NF-L in brain tissue (Table S2) positively correlate with the accumulation of these markers in CSF (Table S1), and may be reflective of motor neuron breakdown (179). Similarly, miR-146a and miR-338-3p, both increased in spinal cord (Table S2), are also detected at a greater level in circulating blood cells of ALS patients (Table S1). These two miRNAs are involved in the regulation of the inflammatory response (197) and the neuromuscular junction (184, 198). In addition, miR-206, a skeletal muscle growth regulator (183), is increased in ALS muscles across studies [Table S1, 2 studies show significant increases (199, 200), the third study only shows a tendency toward an increase in levels (201)]. Together these data reinforce the suggestion that these candidate biomarkers may have utility in determining the status of motor neurons, inflammatory cells and muscle in ALS at different stages of the disease.

When looking at the proteomic and transcriptomic signature of ALS tissues, most observations have not been reproduced across studies. This lack of repeatability could be attributed to numerous factors, such as: different study populations; different types of control subject; different sample sources; different stages of the disease; and the use of different methodological strategies (Table S2).

However, when looking at the different pathways affected in nervous or muscle tissues, we can identify dominant signatures. For instance, skeletal muscle exhibits a dysregulation of pathways involved in muscle atrophy/growth, cytoskeletal maintenance and metabolism, while the central nervous system exhibits inflammatory and excitotoxicity features accompanied by disruptions in axonal transport, cell death, autophagy, metabolism, and RNA processing (Table S2). Concordantly, the systematic decrease of N-acetyl-aspartate observed *in vivo* by magnetic resonance spectrometry in the central nervous system across studies reflects (Table S2) neuron degeneration. These markers likely capture most strongly the endpoints of ALS disease, including degeneration processes in motor neuron death, and muscle denervation and atrophy, and it will be important for future studies to identify biomarkers that track early features of the disease.

## Conclusion

The number of monogenic forms, combined with potential multisystemic contributions to ALS pathology, render it difficult first to unravel physiopathological events, and then to understand which of these events could be pharmacologically targeted. However, by taking a wide-angle view of the pathways affected in different monogenic forms of the disease, it is possible to discern patient strata, with each stratum potentially representing a separate target for therapeutic intervention. Such a strategy is directly applicable to monogenic forms of ALS—known in ~20% of current ALS cases—and future work may discover the extent to which each of these potential targets are transferrable to the 80% of cases in which causal links (genetic or otherwise) have not been identified. Identifying biomarkers to diagnose ALS patients and predict their progression (prognostic biomarkers) may also lead to the identification of patient strata in these non-causally linked forms of ALS.

**Figure 3:**
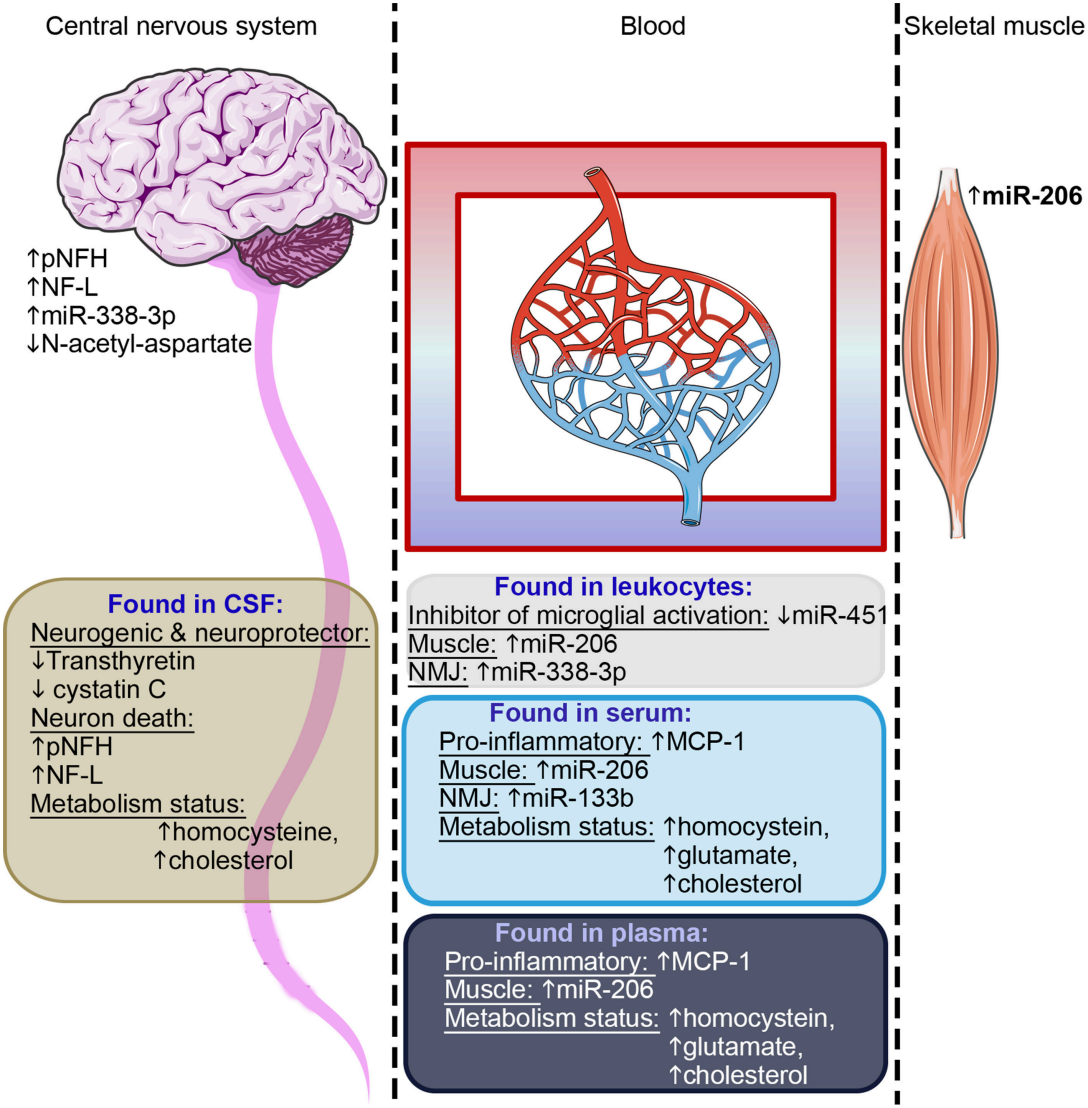
Summary of candidate biomarkers consistently found across studies. Candidates observed in CSF are highlighted in brown, in leukocytes in gray, in serum light blue and in plasma dark blue. These candidate biomarkers reflect the motor neuron health, the inflammatory status, skeletal muscle health, and metabolism status–as indicated in each text block. Some of these candidates were found in postmortem central nervous tissue or on muscle biopsies. NMJ, neuromuscular junction.

Identifying such biomarkers in ALS is a significant challenge as it involves the assessment, not only of motor neuron health status, but also that of other cell types affected in ALS such as astrocytes, microglia, skeletal muscle and inflammatory cells. In this review, we collated across a large number of recently published studies on ALS biomarkers covering several different cell and tissue types (76 studies on body fluids and 42 studies on tissues), and identified only a relatively few candidates that are consistently identified as potential biomarkers across multiple independent studies. These candidate biomarkers are predominantly reflective of motor neuron health, the inflammatory status, and skeletal muscle health (Figure 3). As ALS is increasingly recognized as a multi-systemic disease, it is thus important to track the progression or the recovery of these multiple tissues during clinical trials. In addition, some of these candidates have been confirmed in murine models, e.g., miR-206 in SOD1-G93A mice reflects disease progression in the murine model (202), making them interesting candidates for assessment in pre-clinical studies. As a multi-systemic disease, it is likely that a panel of biomarkers will be needed to fully capture features of ALS pathology.

Considering the different source tissues and the potential implication of each of these in the pathology, our capacity to detect them in accessible fluids, and also the desire to have biomarkers that are confirmed in multiple studies, we would suggest that a useful approach to obtain an overall picture of disease progress in any given patient, may be to combine biomarker candidate molecules from across those listed in Table 2. For example, of biomarkers confirmed in multiple studies, we could suggest a panel of Cystatin C, pNFH and NF-L, all reflecting neuronal survival, MCP1 as a pro-inflammatory marker, the MiRs 206 and 133b reflecting muscle origin and neuromuscular junction, respectively, and some indicators of dysregulated metabolism such as homocysteine, glutamate, or cholesterol. Such a panel (or a variation of it with similarly diverse properties in terms of tissue origin), would be useful to assess the overall “health status” of different tissues. However, all of the biomarkers so far proposed require further validation, as would any specific combination of them.

**Table 2:**
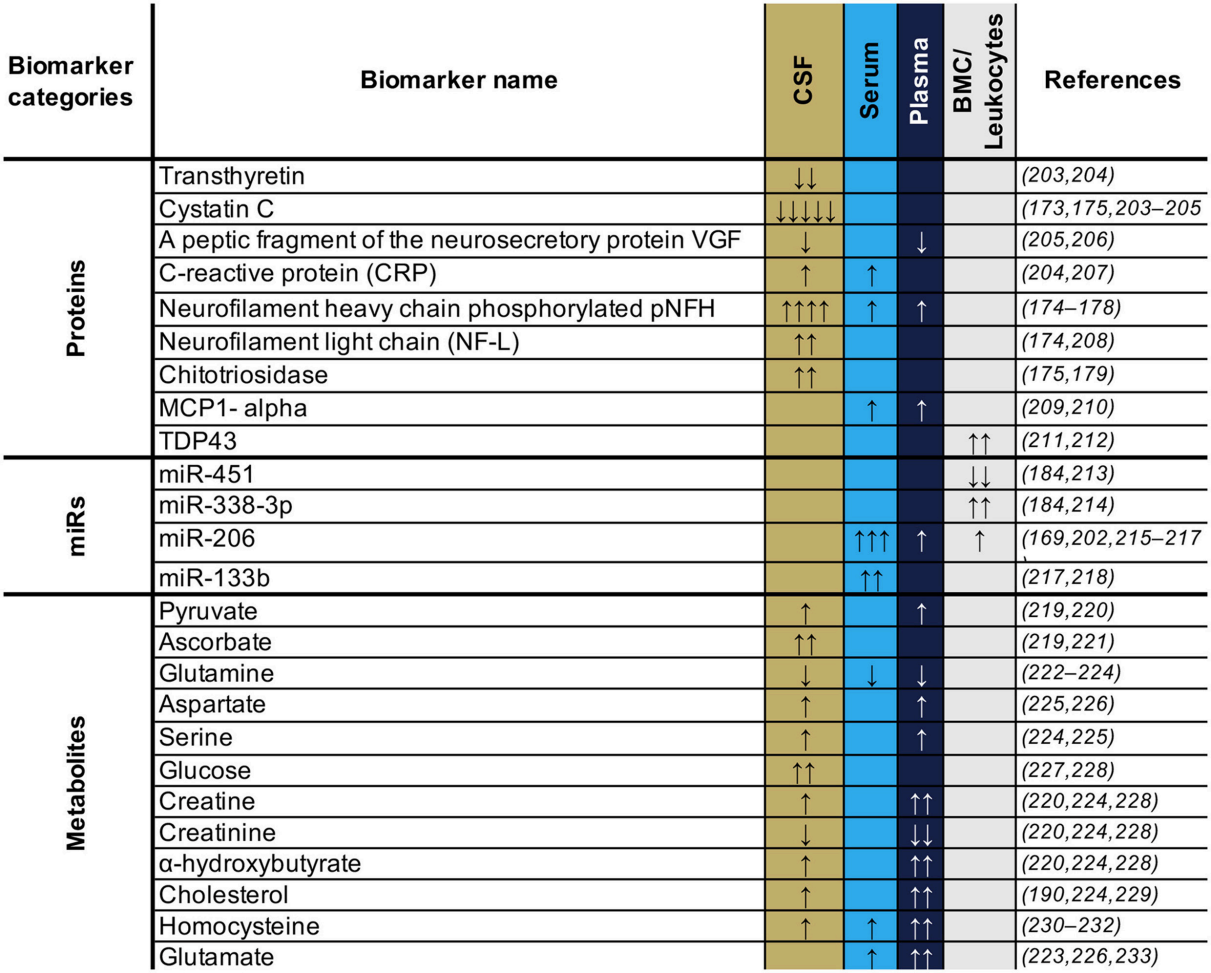
Circulating biomarker candidates consistently observed and confirmed across studies.

*This table is a summary of the detailed Table S1. Data are organized per category of molecule investigated. In each category, the source material is indicated as follows: Gold = CSF, Light blue = Serum, Dark Blue = Plasma, Gray = Blood cells. ↑ = Concentration increased in ALS patients compared to controls, ↓ = Concentration decreased in ALS patients compared to controls. The number of arrows indicates the number of papers describing the increase or decrease of the biomarker considered, in a given tissue. The papers describing these changes are referenced in the last column*.

The development of a heterogeneous multi-biomarker panel—likely including robust new biomarkers and the biomarkers cited in this report—could be seen as a priority, not only for diagnostic purposes but also for prognostic or predictive applications.

## Author Contributions

UV, VM, and MS collated the data from the literature, and wrote the paper. WD and SD organized the data, wrote the paper. AB, WD, and SD edited the paper.

### Conflict of Interest Statement

The authors declare that the research was conducted in the absence of any commercial or financial relationships that could be construed as a potential conflict of interest.
